# The effect of changing pregnancy intentions on preconception health behaviors: a prospective cohort study

**DOI:** 10.1007/s11764-022-01281-1

**Published:** 2022-10-27

**Authors:** Hena Naz Din, David Strong, Savitri Singh-Carlson, Heather L. Corliss, Sheri J. Hartman, Hala Madanat, H. Irene Su

**Affiliations:** 1 Herbert Wertheim School of Public Health and Human Longevity Science, University of California San Diego, 9500 Gilman Dr., La Jolla, CA 92093 USA; 2 School of Public Health, San Diego State University, 5500 Campanile Dr., San Diego, CA 92182 USA; 3 Moores Cancer Center, University of California San Diego, 3855 Health Sciences Dr., La Jolla, CA 92037 USA; 4 School of Nursing, San Diego State University, 5500 Campanile Mall, San Diego, CA 92182 USA; 5 Center for Research On Sexuality and Sexual Health, Institute for Behavioral and Community Health, San Diego State University, 9245 Sky Park Court, Suite 221, San Diego, CA 92123 USA; 6 Division of Research & Innovation, San Diego State University, 5500 Campanile Dr., San Diego, CA 92182 USA; 7 Institute for Behavioral and Community Health, San Diego State University, 9245 Sky Park Court, Suite 221, San Diego, CA 92123 USA; 8 Division of Reproductive Endocrinology and Infertility, University of California San Diego, 9500 Gilman Dr., La Jolla, CA 92093 USA

**Keywords:** Adolescent and young adult cancer, Preconception health, Pregnancy intention, Physical activity, Smoking, Survivorship care

## Abstract

**Purpose:**

Pregnancy intentions are associated with preconception health behaviors but are understudied among female adolescent and young adult (AYA) cancer survivors. Preconception health is critical for survivors because they face unique risks to fertility and pregnancy from late effects of cancer treatments. This study prospectively assessed the effect of pregnancy intention on physical activity (PA) and smoking behaviors among female AYA survivors.

**Methods:**

A cohort of 1049 female AYA survivors were recruited between 2013 and 2017. Participants were 18–39 years and had completed primary cancer treatment. Longitudinal mixed effects analysis was conducted on participants who completed at least 2 of 4 questionnaires over 1.5 years. Two measures were used to capture multiple dimensions of pregnancy intention. The pregnancy intention score (PIS) captured *wanting* and *planning* dimensions and represented a scaled response of low to high intention. The *trying* dimension captured urgent intention and ranged from not trying, ambivalent (neither attempting nor avoiding pregnancy), and trying now. Intention change was assessed between each consecutive time points. Final analysis was conducted with multiple imputations.

**Results:**

Survivors with increased intention measured by *trying* was associated with increased PA over time (adjusted B [95%CI]: 0.3 [0.01, 0.5]) compared to survivors with no changes or decreased *trying* intention. PIS was not significantly associated with preconception behaviors. No measure of intention was associated with smoking behavior.

**Conclusions:**

Increasingly urgent pregnancy intention (*trying* dimension) was associated with higher preconception PA.

**Implications for cancer survivors:**

Screening for immediate intentions can identify AYA survivors in need of early preconception health promotion.

**Supplementary Information:**

The online version contains supplementary material available at 10.1007/s11764-022-01281-1.

## Introduction

Fertility and family planning are key areas of focus for adolescent and young adult cancer (AYA) survivors [1]. Preconception health is critical for survivors because they face unique risks to fertility and pregnancy health due to late effects of cancer treatments, are susceptible to unplanned pregnancies, and are known to engage in unhealthy behaviors (i.e., smoking, binge drinking) [2–4]. Two key modifiable health behaviors during preconception include physical activity (PA) and cigarette smoking [5]. Both have significant effects on maternal and neonatal health, while smoking can also reduce fertility among women [6–8]. Strategies to reduce adverse health behaviors prior to conception in AYA survivors can improve pregnancy health and outcomes.

Pregnancy intentions are associated with health behaviors however these findings are not consistent and methodologically limited [9–11]. Pregnancy intention is a multifaceted concept that represents a spectrum of intended actions to achieve or avoid a pregnancy [12, 13]. Different dimensions of intention like *wanting* a child, *planning* to become pregnant, and *trying* represent levels of urgency to become pregnant and are theorized to be associated with behavior as urgency increases [14]. Additionally, intentions are known to change before and throughout pregnancy as life circumstances change for women [12]. Currently, most studies assess pregnancy intention at one time point, retrospectively, and mainly by the *planning* dimension [11, 15]. In a systematic review of the association between pregnancy intention and health behaviors, Hill et al. (2019) found among 303 studies only 7% evaluated intention prospectively, and most evaluated general levels of pregnancy intention at one time point [11]. Each of these methods can lead to bias in findings. In particular, retrospective assessment of intention can lead to biased results in which, for example, unintended pregnancies are underestimated because wantedness as an intention increases during a pregnancy [15, 16]. Prospective assessment of pregnancy intention with repeated evaluation is needed to better understand the role of pregnancy intention on preconception health behavior.

Collectively, there is limited understanding of how pregnancy intentions may impact female AYA survivors’ preconception behavior, especially longitudinally. Intentions to become pregnant is high among cancer survivors, upwards of 60–78%, and often is high regardless of the type of cancer and treatments experienced [17, 18]. Despite this, most studies with cancer survivors focus on factors associated with unplanned pregnancies or attempt to contextualize why survivors may or may not desire to have children after cancer [19–21]. Only one study evaluated the association between pregnancy intentions and preconception behaviors among female AYA survivors and found intentions during preconception were positively association with PA, but only cross-sectionally [14]. The current study furthers our knowledge by understanding longitudinal associations of changing pregnancy intention on preconception PA and smoking. It is hypothesized that increased pregnancy intentions will be associated with higher engagement in healthy preconception behaviors among AYA cancer survivors.

## Methods

This study used data from the Reproductive Window in Young Adult Cancer Survivors (WINDOW) study, a prospective cohort study to estimate the trajectory of ovarian function among AYA survivors [22]. The State of California Committee for the Protection of Human Subjects and the Institutional Review Boards at the University of California, San Diego, and the Texas Department of State Health Services approved the WINDOW study. Participants were recruited through California and Texas cancer registries, social media, and physician referrals. Eligible participants included females, 18–39 years old, diagnosed with cancer between 15 and 39 years of age, at variable intervals since completing primary cancer treatment, and had at least one ovary. Exclusion criteria were uncontrolled endocrinopathies and multiple cancers or recurrence. Participants were followed for 18 months between 2013 and 2017 and were asked to complete study questionnaires that included assessment of pregnancy intentions and preconception behaviors every 6 months. If participants missed replying to a survey at any follow-up, they were still included and asked to complete surveys at the next study follow-up. For this analysis, participants who completed at least 2 surveys were included. Women who were pregnant or breastfeeding at each time point were excluded.

### Measurements

#### Pregnancy intention

Multiple dimensions of pregnancy intention were assessed by two variables: the Pregnancy Intention Score (PIS) and attempting pregnancy now (*trying* dimension). These specific measures of intention were utilized because they correlate with urgent vs. non-urgent intention based on the Rubicon Action Model [14, 23]. Specifically, PIS is associated with non-urgent intention and the *trying* dimension represents urgent intention. Per the Rubicon Action Model urgency of intention translates to higher likelihood of action [14, 23]. The PIS represents a summed score of *wanting* and *planning* dimensions of pregnancy intention on a 5-point scale ranging from low intention (PIS = 0) (not wanting/planning of a child to wanting) to high intention (PIS = 2) (planning a pregnancy now) [14]. When evaluated for internal consistency, the scale showed good reliability (Cronbach *α* = 0.8).

One item captured the dimension of *trying* where participants reported if they were attempting to become pregnant now. Responses included *yes-trying now*, *no-avoiding pregnancy*, and *neither trying nor avoiding pregnancy*. *Neither* responses were categorized as ambivalent intention as an umbrella term for any reasons for indecision towards pregnancy. Further details on the creation of the PIS and the use of both measures is discussed separately [14].

#### Change in pregnancy intention

Changes in pregnancy intention were captured at each 6-month increment compared to the last time point. Categories included: no change in intention, increased intention, and decreased intention. Numeric changes between 0.5 and 2 in the PIS reflected change in intention. For the *trying* dimension, *not trying* represented lowest pregnancy intention, whereas *trying now* represented highest intention with ambivalent responses in the middle. Any change between these responses, respectively, reflected increasing or decreasing intention. For example, change from *not trying* to ambivalent represented an increase in intention.

#### Physical activity

Participants were asked how many days they were physically active in the past 7 days for at least 30 min/day, including PA that increased heart rate and breathing. This one-item tool from NHANES Physical Activity Questionnaire has test–retest reliability (*r* = 0.72–0.82) in adult and adolescent populations and had modest concurrent validity with objective measures of activity when compared to more comprehensive scales like the Global Physical Activity Questionnaire and Oxford Physical Activity Questionnaire [24–26].

#### Current smoking behavior

Participants were asked if they currently smoke tobacco with final responses as follows: *current smoker* (includes *daily* and *less than daily*) and *non-smoker* [27]. *Don’t know* responses were excluded from analysis.

#### Perceived infertility risk

Participants were asked if they felt their own fertility was greater, same, or less than their female peers [28]. Responses were collapsed to compare any perception of increased risk to no perception of increased risk. Final categories were as follows: *no increased risk* (includes greater or same level of fertility) and *increased risk* (includes less fertile or infertile).

#### Confounders

Demographic covariates included age, race, ethnicity, sexual orientation, education, income, marital status, and health insurance coverage. Respondents ranked their overall general health with 5 responses from excellent to poor. Body mass index (BMI) was calculated with self-reported weight and height. Self-reported comorbidities were categorized as cardiovascular/pulmonary, endocrine, psychological, and other comorbidities. Additional covariates identified as potential confounders included parity and consultation with a fertility specialist before, during, or after cancer treatment. Psychosocial factors included stress measured by the Perceived Stress Scale-10 [29], depression measured by the Patient Health Questionnaire depression scale [30], and social support by RAND institutes medical outcomes study survey [31]. Time since cancer treatment was assessed as a potential confounder.

### Statistical analysis

The exposure was change in pregnancy intention in both PIS and *trying* to become pregnant. Outcomes were days of PA in the last week and current smoking behavior. Covariates were assessed for multicollinearity and reduced if closely associated (Rho >  = 0.5). Remaining covariates were assessed for time variation and if significantly changing overtime, were included as time-varying covariates. All covariates were included and then reduced if non-significant in models and did not present confounding (≤ 10% change in parameter). Frequencies of each variable were described and bivariate tests of association were determined with generalized mixed effects models.

Multivariable mixed effects models, to allow for individual outcome trajectories, were used to model preconception behavior changes. Time was kept categorical within analyses to compare changes over time from baseline. Change in intention was lagged to evaluate outcomes at each consecutive 6-month time point. Thus the first change variable assessed intention change from baseline to 6 months and this was evaluated with behavioral outcomes at the 6-month survey time point. Linear mixed effect models (LMMs) evaluated changes in days of PA and generalized LMMs (GLMMs) modeled changes in smoking status over each survey time point. The ‘lme4’ package in R Studio Version 1.2.5001 was used to analyze both the LMM and GLMMs models [32]. Perceived infertility risk and parity were assessed as effect modifiers in each final parsimonious model as both interaction terms and by stratified analysis.

The main analysis was conducted with multiple imputation (MI) to mitigate reduced power and bias due to attrition [33]. MI estimated missing values using models developed with data from complete cases (participants with no missing data). Missing values were retained at the baseline time point for change in intention variables. MI was conducted in R with the Multiple Imputation Chain Equation (MICE) package and final models were pooled over 60 imputed data sets and summarized [34]. Further information on the specifications used for MI in this study is included in Supplementary File 1. Demographic differences were assessed between responders and nonresponders with each behavior model.

## Results

**Fig. 1:**
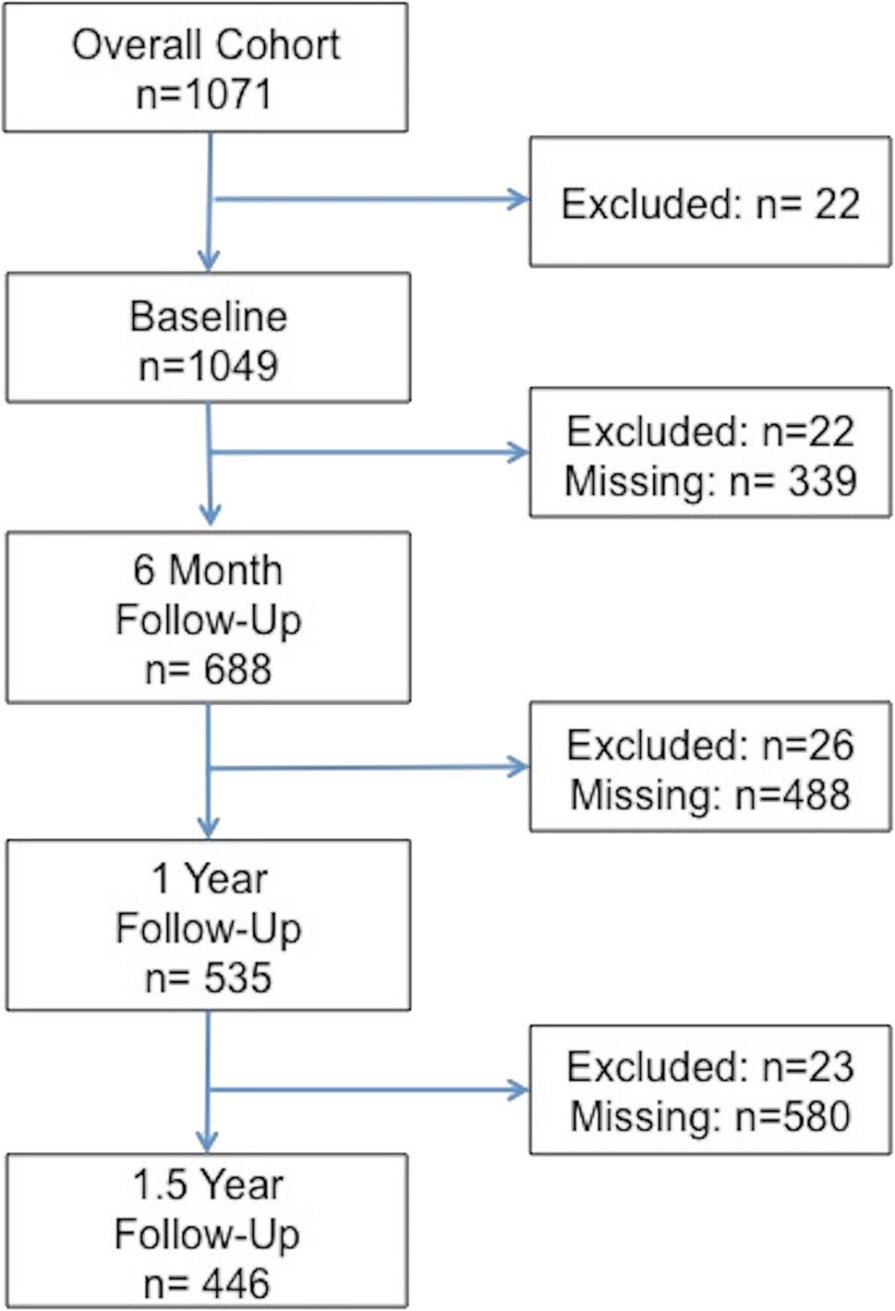
Number of participants at each study time point, who were included in the study cohort, were missing, or were excluded due to an existing pregnancy or reported breastfeeding

More than 30,000 recruitment letters were sent to potentially eligible individuals identified by the California and Texas cancer registries, social media, and physician referrals. Of this group n = 1825 contacted the study team and were assessed for eligibility, 1269 were eligible, and 1159 consented to the study. A total of 1071 eligible participants completed baseline surveys, of which 22 were excluded at baseline because they were either pregnant or breastfeeding. Overall, 65% of the cohort responded to at least 2 surveys and were included in final analyses (Fig. 1). Mean age at cancer diagnosis was 25.7 (standard deviation (SD): 5.8) and mean time to interview from cancer diagnosis was 7.6 (SD: 4.9) years. Baseline characteristics of the cohort are reported in Table 1. At enrollment, most participants were White (74.3%), non-Hispanic (74.8%), partnered (68.8%), and had a mean age of 33 years. Common cancers survived were blood/leukemia/lymphoma (34.9%), breast (22.8%), and skin (18.6), and most participants considered themselves to be at risk of infertility (63.3%). Employment status, household income, and parity were included in models as time-varying covariates.

**Table 1:** Demographic and cancer characteristics of female AYA survivors (*n* = 1049), 2013–2017

Covariates*	
Age at questionnaire (mean (SD))	33.3 (4.9)
Race	
White	776 (74.3)
Black	30 (2.9)
Asian/Native Hawaiian/Native Alaskan/Native Indian	76 (7.3)
Mixed/other race	163 (15.6)
Hispanic ethnicity	265 (25.2)
Heterosexual	992 (92.6)
Married/living with partner	737 (68.8)
≥ College education	763 (71.2)
Employed	815 (76.1)
≥ $51,000 household income	719 (67.1)
≥ 1 Parity	459 (42.9)
Health insurance	1025 (95.7)
BMI	
< 18.5	34 (3.2)
18.5–24.9	457 (42.7)
25–29.9	244 (22.8)
≥ 30	302 (28.2)
General health	
Excellent	100 (9.3)
Very Good	410 (38.3)
Good	429 (40.1)
Fair	115 (10.7)
Poor	14 (1.3)
≥ 1 comorbidities	810 (75.6)
Stress	
No/low stress	391 (36.5)
Moderate stress	596 (55.6)
High stress	84 (7.8)
Depression	
No significant depression (0–4)	512 (47.8)
Mild (5–9)	295 (27.5)
Moderate (10–14)	158 (15.8)
Severe (15–24)	95 (8.9)
Social support (mean(SD))	4.2 (0.9)
Cancer type	
Breast	244 (22.8)
Blood/leukemia/lymphoma	374 (34.9)
Thyroid	120 (11.2)
Reproductive (cervix, uterus, ovary)	28 (2.6)
Gastrointestinal	74 (6.9)
Bone/soft tissue	32 (3.0)
Skin	199 (18.6)
Ever visited fertility specialist	294 (27.5)
Increased perceived Infertility Risk	678 (63.3)

*Variables depicted as *n*(%) unless otherwise indicated

**Fig. 2:**
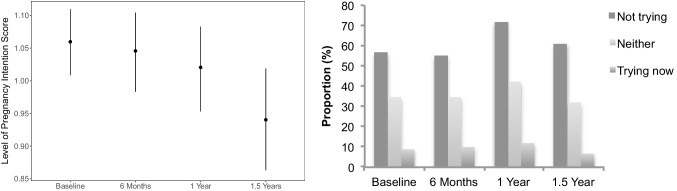
Sample mean and 95% CI of the pregnancy intention score (left) and proportions of the trying pregnancy intention (right) over time

Longitudinal variation in pregnancy intention was observed both within individuals (data not shown) and over time for the overall cohort (Fig. 2). Only 25% and 17% of participants reported the same level of PIS and *trying* intention at each follow-up time point, respectively. Mean PIS and proportion of *trying* to become pregnant significantly reduced over time in Asian/Native Hawaiian/Alaskan/Indian groups and differed by BMI, stress, and perceived infertility risk (Supplementary File 2). Only PIS increased among parous participants.

### Physical activity

Over time, participants reported significantly less PA (Supplementary File 2). Pooled estimates from MI models reflected increased *trying* intention was associated with increased PA over time (adjusted B [95%CI]: 0.3 [0.01, 0.5]) compared to participants with no changes in intention (Table 2). Participants with decreased intention did not differ significantly in PA from participants with no change in intention (adjusted B [95%CI]: 0.2 [− 0.1, 0.5]). Adjusted analysis with complete cases saw similar significant association with increased *trying* dimension associated with increased PA (B 0.2 [0.04, 0.32]) (Table 2). Changes in PIS intention in both MI and complete case models was not associated with PA over assessments (Table 2). Post hoc analysis describing missing data patterns compared participant demographics with PA responses vs. those missing any PA data showed a higher proportion of missing participants were Hispanic, had less than a college education, and, at later time points, were less likely to be White and more likely to be mixed/other race (*p* < 0.005). Race, ethnicity, and education were retained in all final evaluative models along with other covariates for adjustment.

**Table 2:** Mixed effects models of the association of changes in pregnancy intention score (PIS) (left) and trying to become pregnant (right) with physical activity and smoking

	Physical activity				Smoking			
	PIS^a^		Trying^b^		PIS^c^		Trying^d^	
	Adjusted (95% CI)	*p*	Adjusted (95% CI)	*p*	Odds ratio (95% CI)	*p*	Odds ratio (95% CI)	*p*
Multiple imputation model-fixed effects								
No change in intention	Reference	-	Reference	-	References		References	
Decreased intention	0.6 (− 0.2, 0.3)	0.6	0.2 (− 0.1, 0.5)	0.12	1.5 (0.62, 3.56)	0.4	1.67 (0.78, 3.53)	0.19
Increased intention	0.2 (− 0.1, 0.4)	0.2	0.3 (0.01, 0.5) 0	0.04	1.35 (0.69, 3.56)	0.5	1.06 (0.41, 2.80)	0.89
Random effects	1.7		1.7		7.8		5.4	#increase 0.06
Complete cases model- fixed effects								
No change in intention	Reference	**-**	Reference	-	References		References	
Decreased intention	0.001 (− 0.1, 0.1)	0.9	0.1 (− 0.05, 0.23)	0.2	2.5 (0.66, 9.31)	0.2	3.3 (0.8, 13.5)	0.1
Increased intention	0.14 (− 0.1, 0.29)	0.05	0.2 (0.04, 0.32)	0.01	1.1 (0.20, 5.78)	0.9	0.5 (0.1, 4.5)	0.5
Random effects	1.6		1.7		3.3		3.3	

^a^Model adjusted for time, race, ethnicity, age at baseline, education, BMI, general health, stress, social support, perceived infertility risk

^b^Model adjusted for time, race, ethnicity, age at baseline, education, employment, household income, BMI, general health, stress, perceived infertility risk

^c^Model adjusted for time, age at enrollment, race, ethnicity, education, marital status, employment, income, BMI, general health, presence of insurance, stress, social support, comorbidities, parity, perceived infertility risk

^d^Model adjusted for time, race, ethnicity, employment, income, perceived infertility risk

### Current smoking behavior

The proportion of current smokers reduced among participants over time (Supplementary File 2). In both pooled estimates from MI models and complete case models no significant differences were found between those with changing intentions (decreased or increased) compared to participants with no change in intention (Table 2). Post hoc analysis of predictors of missingness showed a higher proportion of cases with missing smoking status reported ambivalent intention and attempting pregnancy now, were Hispanic, and of a lower household income (*p* < 0.005). Ethnicity, and income were retained in all final evaluative models along with other covariates for adjustment.

In each model of PA and smoking, evaluation of two-way interaction terms between perceived infertility risk and PIS or *trying* intentions did not support effect modification in either pooled MI models or complete case analyses. When stratified by perceived infertility risk, decreased *trying* intention was associated with higher odds (2.5 [1.2, 5.7]) of smoking among participants who perceived fertility risk, while PIS was not associated with PA or smoking in either stratum in pooled MI models (Supplementary File 2 Table 5). Two-way interaction terms between parity and PIS or *trying* did not show effect modification in either pooled MI models or complete case analysis. When stratified by parity, effect modification was seen as increased PIS (adjusted 0.6 [95%CI: (0.1, 1.1)]) and trying intention (adjusted 0.3 [95% CI 0.03, 0.5]) was associated with PA in parous but not nulliparous participants (Supplementary File 2 Table 6).

## Discussion

Preconception is a significant period for reproductive-aged women, especially for AYA survivors who may experience greater infertility and perinatal risks [35]. Healthy behaviors during this period can increase the likelihood of a healthy pregnancy and positive neonatal outcomes. Previous studies found positive associations between pregnancy intention and health behaviors among general populations of reproductive aged women, but few explored relationships in AYA survivors and many were methodologically limited [9, 11]. This longitudinal study found that women who began to attempt pregnancy reported higher PA, compared to women with no change in intention. Pregnancy intentions measured by the PIS, or non-urgent intention, were not associated with behavior changes and no measure of intention was associated with smoking behavior. Taken together, urgent intention to become pregnant influences engagement in preconception PA and can be utilized to screen and identify survivors receptive to preconception support and intervention. 

Increasing intention measured by *trying* to become pregnant was associated with preconception PA. Although some studies have found higher PA among intended pregnancies [36, 37], one study found that after controlling for maternal variables like BMI and education, differences in PA by *planning* intention were no longer significant [38]. Here the measurement of intention may explain conflicting findings. *Trying* represents an urgent intention and was hypothesized to be more likely to impact behavior based on behavioral theories [14, 23]. The dimensions of pregnancy intention captured within PIS (*wanting* and *planning)* are generally considered attitudinal intentions, not behavioral intentions [12]. Attitudinal intentions are informational and play a role in intention development however, our study indicates when considering behavior change, measures of urgent intention are more robust. *Trying* as a dimension is not commonly used in intention studies, in contrast to *planning* or *want* intentions. Both the *London Measure of Unplanned Pregnancy* (*LMUP*), considered a gold standard of pregnancy intention measures, and the *One Key Question (OKO)*, a validated measure widely used in clinical settings, evaluate intention primarily from the *planning* and *want* perspectives, respectively [39, 40]. Stratified analysis suggested that PIS and trying were associated with PA among parous survivors (but not among nulliparous survivors); findings require future replication as nulliparous women are more likely to report more preconception PA compared to parous women in general populations [41, 42]. In the context of behavior change in the AYA cancer survivor population, our findings highlight the utility of urgent measures of intention like *trying*.

This study did not find any association between changing pregnancy intentions and current smoking behaviors. Stratified analysis suggested that participants with perceived infertility risk may be more likely to smoke when they do not intend to try to become pregnant, but results need replication because the sample size of those who did not perceive infertility risk was small. Selection bias may have limited these findings as only 6% (*n* = 64) of the baseline cohort reported smoking when other studies have reported higher proportions of female AYA survivors smoke (27–29%) [2, 43]. However, smoking in general is a difficult behavior to modify and most women do not cease smoking till a pregnancy is recognized [44, 45]. Most studies in general populations of women have found no association between intention and smoking behavior, only one study found ambivalent intention was associated with increased smoking behavior [46]. Because pregnancy intention was not associated with smoking behavior, screening for pregnancy intention would likely not tailor smoking cessation discussions for AYA survivors. Different avenues of intervention are needed because AYA survivors do experience unexpected pregnancies and may be exposed to harmful effects of smoking during a sensitive period.

This study made use of MI to retain power in analysis. This study saw an overall 58% loss to follow-up, which is common for prospective cohort studies [47]. Compared to responders, nonresponse was found to be highest among those of Hispanic ethnicity, non-white race, lower income, and lower education. Nonresponse is known to be higher among individuals in these demographics [48–50]. In our study we hypothesized data was *missing at random* (MAR) which assumes missing data or nonresponse is associated only with observed data and not with unobserved data [47]. MAR gives validity to MI because variables predictive of missingness (i.e., Hispanic ethnicity, non-white race) are included in MI estimations and allows for greater accuracy in estimation [51]. Because demographic variables were identified that could estimate likelihood of nonresponse, MAR was a valid assumption for our study and supported robust MI estimations. Additionally, results did not differ between complete case and MI models indicating MI provided greater accuracy in our estimations without adding bias.

A key strength of this study is the prospective evaluation of changing pregnancy intentions every 6 months during the preconception period among AYA survivors. Fluctuation is a characteristic of pregnancy intention because it follows a constructivist formation; situation, environment, time, among many other variables, influence and contribute to the formation of intention [52]. As these external variables change so may intention. Martial status/having a partner, employment, and household income are shown to contribute to changes in pregnancy intentions [53]. During preconception, we saw an overall decrease in intentions whereas previous studies primarily assessed intention change from preconception to post-partum, and here intention increases over time [16, 54]. Given the mean age of our cohort, increasing age may be a reason for a downward trend in intention. Older women, especially women 35 + , are more likely to report lower reproductive intentions among general populations and cancer survivors [55, 56]. This aligns with the constructivist model as pregnancy intention is constantly being reassessed as life circumstances change.

A limitation in our study included the lack of assessment of AYA survivor knowledge on preconception health and healthy behaviors. Knowledge may impact preconception behaviors and would have identified gaps and areas of intervention. Additionally, this study only assessed 2 modifiable behaviors whereas additional preconception behaviors like managing chronic health conditions may be particularly important for AYA survivors who often have co-morbidities and would benefit from guidance on successful management.

## Conclusion

This study furthers our understanding of changing pregnancy intentions and the role of these changes on preconception behaviors among female reproductive-age AYA cancer survivors. Urgent dimensions of pregnancy intention are associated with PA behavior and repeated assessments of intention strengthen findings by capturing changes in pregnancy intention during preconception. Early preconception education and intervention can help women navigate family planning and achieve healthy pregnancies. National guidelines highlight the role of health care providers in guiding family planning, providing education and health promotion during clinic visits [6]. Incorporation of urgent pregnancy intention screening in survivorship care can help facilitate early preconception health promotion and education.

### Supplementary Information

Below is the link to the electronic supplementary material.

Supplementary file1 (DOCX 31 KB)

Supplementary file2 (DOCX 81 KB)
