# Supplementary material for: The effect of changing pregnancy intentions on preconception health behaviors: a prospective cohort study
Source: J Cancer Surviv. 2022 Oct 27;17(6):1660–8. doi: 10.1007/s11764-022-01281-1 (PMC10539193; doi:10.1007/s11764-022-01281-1)
Supplement: Supplementary file 1 — Supplementary file1 (DOCX 31 KB) [file 11764_2022_1281_MOESM1_ESM.docx]

**Title: The effect of changing pregnancy intentions on preconception health behaviors: a prospective cohort study**

**Journal: Journal of Cancer Survivorship**

**Authors & Affiliations:**

**Hena Naz Din, MPH**^1,2^, **David Strong, PhD**^1,3^, **Savitri Singh-Carlson, RN, PhD, FAAN** ^4^, **Heather L. Corliss, MPH, PhD** ^2,5^, **Sheri J. Hartman, PhD** ^1,3^, **Hala Madanat, PhD** ^2,6,7^, **H. Irene Su, MD, MSCE** ^8^

Corresponding Author: Hena Naz Din, Email: [hena.n.din@gmail.com](mailto:hena.n.din@gmail.com), Telephone: 858-735-2406

1. Herbert Wertheim School of Public Health and Human Longevity Science, University of California San Diego. 9500 Gilman Dr. La Jolla, CA. 92093
2. School of Public Health, San Diego State University. 5500 Campanile Dr. San Diego, CA. 92182
3. Moores Cancer Center, University of California San Diego. 3855 Health Sciences Dr. La Jolla, CA. 92037
4. School of Nursing, San Diego State University. 5500 Campanile Mall, San Diego. CA. 92182
5. Center for Research on Sexuality and Sexual Health, Institute for Behavioral and Community Health, San Diego State University. 9245 Sky Park Court. Suite 221. San Diego, CA. 92123
6. Division of Research & Innovation, San Diego State University. 5500 Campanile Dr. San Diego, CA. 92182
7. Institute for Behavioral and Community Health, San Diego State University. 9245 Sky Park Court. Suite 221. San Diego, CA. 92123
8. Division of Reproductive Endocrinology and Infertility, University of California San Diego. 9500 Gilman Dr. La Jolla, CA. 92093

**Methods on Multiple Imputations:**

Multiple imputation was conducted through the R package Multiple Imputation Chain Equation (MICE) package. MICE was ran with fully conditional specification (FCS) modeling which runs iterative regressions of a variable’s values to estimate a distribution of the observed values.^1^ This distribution is then utilized to estimate missing values. Each model was ran with 60 iterations based on the largest value of missingness within the dataset. A missing at random assumption was made for the analysis because difference between responders and nonresponders were captured by observed characteristics and there were no theoretical underlying reasons for missingness base on nonobserved data.^2^ To improve estimation of missing values covariates that may predict missingness and were to be included in final analyses were included in MI models.^2^ By including these covariates relationships between variables are preserved which help in estimation.

Within MICE coding, classification and regression trees (CART) method was used because it works well with continuous and categorical data, preserves the value range in which missing data can be estimated, and does not rely on parametric assumptions for imputation.^3^ This was important so that predicted values of categorical variables remain within appropriate categories.

| **Table 1.** Proportions of missingness and complete cases across independent and outcome variables | | | | |
| --- | --- | --- | --- | --- |
|  | **Cases with Missing Data** | | | **Complete Cases** |
|  | **6 Months**  n(%) | **1 Year**  n(%) | **1.5 Years**  n(%) | **n(%)** |
| **Independent Variables** |  |  |  |  |
| PIS | 409 (38.4) | 553 (51.9) | 663 (62.3) | 338 (31.7) |
| Trying | 377 (35.4) | 530 (49.8) | 642 (60.2) | 389 (36.5) |
| **Outcomes** |  |  |  |  |
| PA | 377 (35.4) | 530 (49.8) | 642 (60.2) | 389 (36.5) |
| Smoking | 381 (35.8) | 535 (50.2) | 643 (60.4) | 382 (35.9) |

References:

1. Oakes JM, Rossi PH. The measurement of SES in health research: Current practice and steps toward a new approach. *Soc Sci Med*. 2003;56(4):769-784. doi:10.1016/S0277-9536(02)00073-4

2. Sterne JAC, White IR, Carlin JB, et al. Multiple imputation for missing data in epidemiological and clinical research: Potential and pitfalls. *BMJ*. 2009;339(7713):157-160. doi:10.1136/bmj.b2393

3. Burgette LF, P. RJ. Multiple Imputation for Missing Data via Sequential Regression Trees. *Pract Epidemiol*. 2010;172(9):1070-1076.
