# Supplementary material for: The effect of changing pregnancy intentions on preconception health behaviors: a prospective cohort study
Source: J Cancer Surviv. 2022 Oct 27;17(6):1660–8. doi: 10.1007/s11764-022-01281-1 (PMC10539193; doi:10.1007/s11764-022-01281-1)
Supplement: Supplementary file 2 — Supplementary file2 (DOCX 81 KB) [file 11764_2022_1281_MOESM2_ESM.docx]

**Appendix Table 1. Mean Pregnancy Intention Score (PIS) (standard deviation) over time by participant characteristics**

| **Covariates** | **Baseline**  n= 1049 | **6 Months**  n= 688 | **1 Year**  n= 535 | **1.5 Years**  n= 446 |
| --- | --- | --- | --- | --- |
|  | mean (SD) | mean (SD) | mean (SD) | mean (SD) |
| **Overall Cohort** | 3.1 (1.5) | 3.1 (1.6) | 3.0 (1.6) | 2.9 (1.6) |
| **Race-** White | 3.1 (1.5)^a^ | 3.0 (1.6) | 3.0 (1.6) | 2.8 (1.6) |
| Black | 3.6 (1.4) | 3.6 (1.4) | 4.1 (0.7) | 3.8 (1.2) |
| Asian/Native Hawaiian/Alaskan/Indian* | 3.6 (1.4) | 3.7 (1.2) | 3.4 (1.4) | 3.0 (1.6) |
| Mixed/Other race | 3.0 (1.6) | 3.1 (1.6) | 3.1 (1.6) | 2.9 (1.7) |
| **Hispanic ethnicity-** Non-Hispanic | 3.1 (1.5) | 3.1 (1.6) | 3.0 (1.6) | 2.9 (1.6) |
| Hispanic | 3.1 (1.5) | 3.1 (1.5) | 2.9 (1.6) | 2.8 (1.6) |
| **Education*-** High school or less | 3.0 (1.5) | 2.8 (1.6)^a^ | 2.7 (1.5)^a^ | 2.6 (1.6) |
| ≥ College education | 3.2 (1.5) | 3.2 (1.6) | 3.1 (1.6) | 2.9 (1.6) |
| **Marital Status-** Not married/ partnered | 3.2 (1.3) | 3.1 (1.3) | 3.2 (1.3) | 3.1 (1.5) |
| Married/ partnered | 3.1 (1.6) | 3.1 (1.7) | 2.9 (1.7) | 2.8 (1.7) |
| **Employment-** Unemployed | 3.0 (1.5) | 3.0 (1.6) | 3.1 (1.6) | 2.8 (1.6) |
| Employed | 3.2 (1.5) | 3.1 (1.6) | 3.0 (1.6) | 2.9 (1.6) |
| **Household Income**  < $51,000 | 3.3 (1.4) | 3.2 (1.4) | 3.1 (1.4) | 3.1 (1.5) |
| ≥ $51,000 | 3.1 (1.6) | 3.1 (1.6) | 3.0 (1.7) | 2.8 (1.7) |
| **BMI*-** <18.5 | 3.2 (1.4) | 3.0 (1.7) | 2.9 (1.7) ^a^ | 2.8 (1.8) |
| 18.5-24.9 | 3.2 (1.5) | 3.2 (1.5) | 3.2 (1.5) | 2.9 (1.6) |
| 25-29.9 | 3.1 (1.6) | 3.2 (1.7) | 3.1 (1.7) | 2.9 (1.7) |
| ≥30 | 3.0 (1.6) | 2.9 (1.6) | 2.7 (1.6) | 2.8 (1.6) |
| **General Health-** Excellent | 3.1 (1.7) | 2.9 (1.6) | 3.0 (1.6) | 2.9 (1.7) |
| Very Good | 3.1 (1.6) | 3.2 (1.6) | 3.1 (1.6) | 2.9 (1.7) |
| Good | 3.1 (1.5) | 3.1 (1.5) | 3.1 (1.5) | 2.9 (1.5) |
| Fair | 3.1 (1.6) | 3.1 (1.6) | 2.9 (1.6) | 2.5 (1.8) |
| Poor | 3.0 (1.5) | 2.5 (1.6) | 2.5 (1.6) | 2.5 (2.1) |
| **Health Insurance-** No insurance | 3.3 (1.5) | 3.2 (1.5) | 3.3 (1.6) | 3.0 (1.8) |
| Has insurance | 3.1 (1.5) | 3.1 (1.6) | 3.0 (1.6) | 2.9 (1.6) |
| **Stress* -** No/low stress | 3.0 (1.6) | 3.1 (1.6) | 3.0 (1.6) | 2.9 (1.6) |
| Moderate stress | 3.2 (1.5) | 3.1 (1.6) | 3.1 (1.5) | 2.9 (1.6) |
| High stress | 3.1 (1.4) | 2.9 (1.7) | 3.0 (1.6) | 2.6 (1.6) |
| **Presence of cardiopulmonary comorbidities-** No | 3.1 (1.5) | 3.1 (1.6) | 3.0 (1.6) | 2.9 (1.6) |
| Yes | 3.1 (1.5) | 3.3 (1.5) | 3.2 (1.5) | 3.0 (1.6) |
| **Presence of psychological comorbidities-** No | 3.2 (1.5) | 3.1 (1.6) | 3.0 (1.6) | 2.8 (1.6) |
| Yes | 3.0 (1.5) | 2.9 (1.6) | 3.0 (1.5) | 3.0 (1.6) |
| **Presence of endocrine comorbidities-** No | 3.2 (1.5) | 3.1 (1.6) | 3.1 (1.6) | 2.9 (1.6) |
| Yes | 3.0 (1.6) | 3.1 (1.6) | 2.9 (1.6) | 2.8 (1.6) |
| **Presence of other comorbidities-** No | 3.1 (1.6) | 3.2 (1.6) | 3.1 (1.6) | 3.1 (1.6) ^a^ |
| Yes | 3.1 (1.5) | 3.0 (1.6) | 2.9 (1.6) | 2.6 (1.6) |
| **Parity*-** None | 3.5 (1.3)^a^ | 3.1 (1.6)^a^ | 3.0 (1.6) | 2.9 (1.6) |
| ≥1 Parity | 2.6 (1.6) | 4.8 (0.5) | 3.3 (2.0) | 3.2 (1.5) |
| **Perceived Infertility Risk*-** No increased risk | 2.8 (1.6)^a^ | 2.7 (1.6)^a^ | 2.8 (1.6)^a^ | 2.4 (1.6) ^a^ |
| Increased risk | 3.3 (1.5) | 3.3 (1.5) | 3.2 (1.5) | 3.2 (1.6) |
| *Significant difference in mean PIS across time points (p <0.05)  ^a^Significant difference in mean PIS between covariate groups at applicable time point (p <0.05) | | | | |

**Appendix Table 2. Distribution of attempting pregnancy (*trying* dimension) over time by participant characteristics**

| **Covariates**^§^ | **Baseline** | | | **6 Month** | | | **1 Years** | | | **1.5 Years** | | |
| --- | --- | --- | --- | --- | --- | --- | --- | --- | --- | --- | --- | --- |
|  | Not Trying  n= 590 | Ambivalent  n= 364 | Trying Now  n= 95 | Not Trying  n=385 | Ambivalent  n=235 | Trying Now  n=68 | Not Trying  n=308 | Ambivalent  n=177 | Trying Now  n=50 | Not Trying  n=261 | Ambivalent  n=135 | Trying Now  n=27 |
| **Age at questionnaire*** (mean (SD)) | 32.8(5.0) | 33.8(4.9) | 34.3(4.1)^a^ | 32.8(5.0) | 33.7(4.7) | 34(3.5) | 32.9(4.9) | 33.5(5.0) | 33.9(3.2) | 33(4.9) | 33.3(4.8) | 32.9(3.5) |
| **Race-** White | 446 75.6) | 249(68.4) | 66(69.5) | 297(77.1) | 154(65.5) | 47(69)^a^ | 244(79.2) | 10(62.1) | 31(62)^a^ | 193(73.9) | 94(69.6) | 19(70.4) |
| Black | 11(1.9) | 16(4.4) | 1(1.1)^a^ | 8(2.1) | 9(3.8) | 3(4.4) | 5(1.6) | 4(2.3) | 3(6) | 5(1.9) | 4(3) | 0(0) |
| Asian/Native Hawaiian/Alaskan/Indian* | 33(5.6) | 30(8.2) | 11(11.6) | 23(6) | 21(8.9) | 3(4.4) | 17(5.5) | 16(9) | 6(12) | 19(7.3) | 12(8.9) | 3(11.1) |
| Mixed/Other race* | 84(14.2) | 60(16.5) | 16(16.8) | 43(11.2) | 45(19.1) | 14(20.6) | 30(9.7) | 40(22.6) | 8(16) | 32(12.3) | 19(14.1) | 4(14.8) |
| **Hispanic ethnicity*-** Non-Hispanic | 447(75.8) | 256(70.3) | 64(67.4)^a^ | 306(79.5) | 174(74) | 49(72.1) | 241(78.2) | 128(72.3) | 39(78) | 200(76.6) | 104(77) | 23(85.2) |
| Hispanic | 129(21.9) | 105(28.8) | 28(29.5) | 65(16.9) | 58(24.7) | 17(25) | 52(16.9) | 44(24.9) | 9(18) | 46(17.6) | 28(20.7) | 3(11.1) |
| **Education-** High school or less | 132(22.4) | 146(40.1) | 28(29.5)^a^ | 66(17.1) | 72(30.6) | 10(14)^a^ | 45(14.6) | 52(29.4) | 5(10) ^a^ | 40(15.3) | 32(23.7) | 5(18.5) |
| ≥ College education | 458(77.6) | 218(59.9) | 67(70.5) | 312(81) | 162(68.9) | 57(83.8) | 255(82.8) | 122(68.9) | 43(86) | 212(81.2) | 101(74.8) | 21(77.8) |
| **Marital Status*-** Not married/ partnered | 197(33.4) | 133(36.5) | 3(3.2.)^a^ | 124(32.2) | 94(40) | 1(1.5) ^a^ | 95(30.8) | 77(43.5) | 2(4) ^a^ | 85(32.6) | 50(37) | 4(14.8) |
| Married/ partnered | 393(66.6) | 231(63.5) | 92(96.8) | 254(66) | 140(59.6) | 66(97.1) | 205(66.6) | 97(54.8) | 46(92) | 167(64) | 83(61.5) | 22(81.5) |
| **Employment-** Unemployed | 131(22.2) | 88(24.2) | 18(18.9) | 86(22.3) | 50(21.3) | 9(13.2) | 70(22.7) | 35(19.8) | 6(12) | 40(15.3) | 29(21.5) | 3(11.1) |
| Employed | 454(76.9) | 270(74.2) | 77(81.1) | 290(75.3) | 180(76.6) | 58(85.3) | 228(74) | 135(76.3) | 42(84) | 219(83.9) | 106(78.5) | 24(88.9) |
| **Household Income-**  < $51,000 | 138(23.4) | 122(33.5) | 19(20)^a^ | 75(19.5) | 67(28.5) | 9(13.2)^a^ | 63(20.5) | 55(31.1) | 5(10) ^a^ | 52(19.9) | 39(28.9) | 2(7.4) ^a^ |
| ≥ $51,000 | 419(71) | 212(58.2) | 72(75.8) | 283(73.5) | 150(63.8) | 55(80.9) | 229(74.4) | 111(62.7) | 42(84) | 199(76.2) | 88(65.2) | 23(85.2) |
| **BMI*-** <18.5 | 16(2.7) | 13(3.6) | 4(4.2)^a^ | 7(1.8) | 11(4.7) | 1(1.5) ^a^ | 7(2.3) | 6(3.4) | 3(6) | 5(1.9) | 5(4.4) | 1(3.7) |
| 18.5-24.9 | 274(46.4) | 134(36.8) | 38(40) | 180(46.8) | 102(43.4) | 29(42.6) | 137(44.5) | 80(45.2) | 21(42) | 116(44.4) | 57(42.2) | 12(44.4) |
| 25-29.9 | 140(23.7) | 81(22.3) | 18(18.9) | 94(24.4) | 40(17) | 21(30.9) | 80(26) | 29(16.4) | 14(28) | 67(25.7) | 24(17.8) | 6(22.2) |
| ≥30 | 142(24.1) | 125(34.3) | 30(31.6) | 89(23.1) | 73(31.1) | 13(19.1) | 70(22.7) | 53(29.9) | 8(16) | 60(23) | 40(29.6) | 6(22.2) |
| **General Health*-** Excellent | 54(9.2) | 34(9.3) | 9(9.5)^a^ | 44(11.4) | 22(9.4) | 7(10.3) | 37(12) | 17(9.6) | 4(8) | 26(10) | 11(8.1) | 5(18.5) |
| Very Good | 242(41) | 119(32.7) | 35(36.8) | 163(42.3) | 85(36.2) | 28(41.2) | 122(39.6) | 67(37.9) | 20(40) | 106(40.6) | 53(39.3) | 8(29.6) |
| Good | 241(40.8) | 150(41.2) | 35(36.8) | 140(36.4) | 99(42.1) | 25(36.8) | 114(37) | 72(40.7) | 18(36) | 100(38.3) | 56(41.5) | 10(37) |
| Fair | 45(7.6) | 52(14.3) | 16(16.8) | 28(7.3) | 24(10.2) | 7(10.3) | 23(7.5) | 15(8.5) | 6(12) | 19(7.3) | 10(7.4) | 3(11.1) |
| Poor | 7(1.2) | 7(1.9) | 0(0) | 2(0.5) | 4(1.7) | 0(0) | 3(1) | 3(1.7) | 0(0) | 0(0) | 3(2.2) | 0(0) |
| **Health Insurance-** No insurance | 21(3.6) | 19(5.2) | 6(6.3) | 11(2.9) | 10(4.3) | 1(1.5) | 7(2.3) | 5(2.8) | 2(4) | 6(2.3) | 5(3.7) | 1(3.7) |
| Has insurance | 569(96.4) | 345(94.8) | 89(93.7) | 367(95.3) | 224(95.3) | 66(97.1) | 293(95.1) | 169(95.5) | 46(92) | 246(94.3) | 128(94.8) | 25(92.6) |
| **Stress* -** No/low stress | 229(38.8) | 115(31.6) | 36(37.9)^a^ | 167(43.4) | 73(31.1) | 31(45) ^a^ | 127(41.2) | 61(34.5) | 22(44) | 106(40.6) | 46(34.1) | 10(37) |
| Moderate stress | 325(55.1) | 209(57.4) | 52(54.7) | 194(50.4) | 141(60) | 34(50) | 160(51.9) | 99(55.9) | 23(46) | 137(52.5) | 75(55.6) | 15(55.6) |
| High stress | 36(6.1) | 40(11) | 7(7.4) | 17(4.4) | 20(8.5) | 2(2.9) | 13(4.2) | 14(7.9) | 3(6) | 9(3.4) | 12(8.9) | 1(3.7) |
| **Social Support**(mean (SD)) | 4.3(0.8) | 4.1(1.0) | 4.4(0.7) ^a^ | 4.3(0.8) | 4.1(0.9) | 4.5(0.7) | 4.3(0.7) | 4.1(1.0) | 4.4(0.7) | 4.2(0.8) | 4.1(0.9) | 4.3(0.7) |
| **Cardiopulmonary comorbidities*-** No | 501(84.9) | 307(84.3) | 76(80) | 332(86.2) | 199(84.7) | 54(79.4) | 263(85.4) | 143(80.8) | 42(84) | 219(83.9) | 117(86.7) | 21(77.8) |
| Yes | 89(15.1) | 57(15.7) | 19(20) | 46(11.9) | 35(14.9) | 13(19.1) | 37(12) | 31(17.5) | 6(12) | 33(12.6) | 16(11.9) | 5(18.5) |
| **Psychological comorbidities-** No | 427(72.4) | 258(70.9) | 72(75.8) | 282(73.2) | 171(72.8) | 49(72.1) | 231(75) | 131(74) | 35(70) | 195(74.7) | 102(75.6) | 18(66.7) |
| Yes | 163(27.6) | 106(29.1) | 23(24.2) | 96(24.9) | 63(26.8) | 18(26.5) | 69(22.4) | 43(24.3) | 13(26) | 57(21.8) | 31(23) | 8(29.6) |
| **Endocrine comorbidities-** No | 476(80.7) | 295(81) | 70(73.7) | 315(81.8) | 181(77) | 55(80.9) | 250(81.2) | 135(76.3) | 39(78) | 206(78.9) | 104(77) | 22(81.5) |
| Yes | 114(19.3) | 69(19) | 25(26.3) | 63(16.4) | 53(22.6) | 12(17.6) | 50(16.2) | 39(22) | 9(18) | 46(17.6) | 29(21.5) | 4(14.8) |
| **Other comorbidities-** No | 412(69.8) | 228(62.6) | 69(72.6)^a^ | 255(66.2) | 144(61.3) | 50(73.5) | 202(65.6) | 106(59.9) | 33(66) | 161(61.7) | 83(61.5) | 18(66.7) |
| Yes | 178(30.2) | 136(37.4) | 26(27.4) | 123(31.9) | 90(38.3) | 17(25) | 98(31.8) | 68(38.4) | 15(30) | 91(34.9) | 50(37) | 8(29.6) |
| **Parity-** None | 339(57.5) | 208(57.1) | 65(68.4) | 385(100) | 232(98.7) | 66(97) ^a^ | 306(99.4) | 175(98.9) | 48(96) | 256(98.1) | 134(99.3) | 27(100) |
| ≥1 Parity | 251(42.5) | 156(42.9) | 30(31.6) | 0(0) | 3(1.3) | 2(2.9) | 2(0.6) | 2(1.1) | 2(4) | 5(1.9) | 1(0.7) | 0(0) |
| **Perceived Infertility Risk*-** No increased risk | 271(45.9) | 99(27.2) | 11(11.6) ^a^ | 188(48.8) | 59(25.1) | 15(22) ^a^ | 146(47.4) | 53(29.9) | 14(28) ^a^ | 124(47.5) | 38(28.1) | 6(22.2) ^a^ |
| Increased risk | 319(54.1) | 265(72.8) | 84(88.4) | 197(51.2) | 176(74.9) | 53(77.9) | 162(52.6) | 124(70.1) | 36(72) | 137(52.5) | 97(71.9) | 21(77.8) |
| ^§^Variables depicted as n(%) unless otherwise indicated  *Significant difference in proportion reporting attempting pregnancy across time points (p <0.05)  ^a^Significant difference in proportion of attempting pregnancy between covariate groups at applicable time point (p <0.05) | | | | | | | | | | | | |

**Appendix Table 3. Mean physical activity (standard deviation) over time by pregnancy intentions**

| **Covariates** | **Baseline**  n=1049 | **6 Month**  n=688 | **1 Year**  n=535 | **1.5 Years**  n=423 |  |
| --- | --- | --- | --- | --- | --- |
| **Overall Cohort*** | 4.1 (2.0) | 3.9 (2.0) | 3.7 (1.9) | 3.8 (1.9) |  |
| **Pregnancy Intention Score** |  |  |  |  |  |
| 0 (Don’t want and not planning pregnancy) | 4.0 (2.0) | 4.0 (2.1) | 3.8 (2.0) | 3.8 (1.9) |  |
| 0.5 (Don’t want now but planning later) | 4.1 (2.1) | 2.4 (1.4) | 2.9 (1.2) | 4.7 (1.7) |  |
| 1 (Want but not planning pregnancy) | 4.2 (2.2) | 4.4 (2.1) | 3.8 (1.9) | 4.4 (2.4) |  |
| 1.5 (Want and planning later) | 3.9 (1.9) | 3.8 (1.9) | 3.7 (1.9) | 3.8 (1.9) |  |
| 2 (Want and planning now) | 4.5 (1.9) | 4.2 (2.1) | 3.7 (2.0) | 3.9 (1.9) |  |
| **Trying*** |  |  |  |  |  |
| Not trying | 4.0 (1.9) | 3.8 (1.9) | 3.7 (1.8) | 3.8 (2.0) |  |
| Neither | 4.1 (2.1) | 4.0 (2.1) | 3.7 (2.0) | 3.9 (2.0) |  |
| Trying now | 4.6 (1.8) | 4.4 (2.3) | 4.0 (2.0) | 4.1 (2.0) |  |

* Significant difference in PA across time points (p <0.05)

**Appendix Table 4. Distribution of smoking (n (%)) over time by pregnancy intentions**

|  | **Baseline** | | **6 Month** | | **1 Year** | | **1.5 Years** | |
| --- | --- | --- | --- | --- | --- | --- | --- | --- |
|  | Nonsmoker  n=976 | Smoker  n=64 | Nonsmoker  n=656 | Smoker  n=28 | Nonsmoker  n=514 | Smoker  n=16 | Nonsmoker  n= 406 | Smoker  n= 16 |
| **Pregnancy Intention Score** |  |  |  |  |  |  |  |  |
| 0 (Don’t want & not planning) | 280 (28.7) | 26 (40.6) | 203 (30.9) | 10 (35.7) | 168 (32.47) | 4 (25.0) | 155 (38.2) | 4 (25.0) |
| 0.5 (Don’t want now but planning later) | 24 (2.5) | 3 (4.7) | 12 (1.8) | 0 (0) | 12 (2.3) | 0 (0) | 6 (1.5) | 1 (6.2) |
| 1 (Want but not planning) | 87 (8.9) | 8 (12.5) | 55 (8.4) | 5 (17.9) | 46 (8.9) | 5 (31.2) | 32 (7.9) | 3 (18.8) |
| 1.5 (Want and planning later) | 364 (37.3) | 20 (31.2) | 228 (34.8) | 8 (28.6) | 165 (32.1) | 5 (31.2) | 119 (29.3) | 5 (31.2) |
| 2 (Want and planning now) | 177 (18.4) | 7 (10.9) | 127 (19.4) | 4 (14.3) | 101 (19.6) | 2 (12.5) | 73 (18.0) | 3 (18.8) |
| **Trying** |  |  |  |  |  |  |  |  |
| Not trying | 562 (57.6) | 28 (43.8)^a^ | 372 (56.7) | 12 (42.9) | 299 (58.2) | 8 (50.0) | 251 (61.8) | 9 (56.2) |
| Neither | 324 (33.2) | 34 (53.1) | 219 (33.4) | 15 (53.6) | 166 (32.3) | 7 (43.8) | 129 (31.8) | 6 (37.5) |
| Trying now | 90 (9.2) | 2 (3.1) | 65 (9.9) | 1 (3.6) | 49 (9.5) | 1 (6.2) | 26 (6.4) | 1 (6.2) |
| *Significant difference in smoking across time points (p <0.05)  ^a^Significant difference in smoking between groups at applicable time point (p <0.05) | | | | | | | | |

**Appendix Table 5.** Mixed effects models of the association of changes in pregnancy intention score (PIS) (left) and trying to become pregnant (right) with physical activity and smoking stratified by perceived infertility risk

|  | **Physical Activity** | | | | **Smoking** | | | |  |
| --- | --- | --- | --- | --- | --- | --- | --- | --- | --- |
|  | **PIS^a^** | | **Trying^b^** | | **PIS^c^** | | **Trying^d^** | |  |
|  | Adjusted  B(95% CI) | p | Adjusted  B(95% CI) | p | Odds Ratio  (95% CI) | p | Odds Ratio  (95% CI) | p | |
| **No perceived infertility risk** | | | | | | | | |  |
| **Multiple Imputation Model**- Fixed Effects |  |  |  |  |  |  |  |  | |
| No Change in Intention | Reference | - | Reference | - | References |  | References |  | |
| Decreased Intention | 0.2 (-0.2, 0.7) | 0.3 | 0.02 (-0.5, 0.5) | 0.9 | 1.7 (0.3, 8.5) | 0.5 | 0.3 (0,0) | 1 | |
| Increased Intention | 0.4 (-0.03, 0.9) | 0.06 | 0.2 (-0.2, 0.6) | 0.3 | 2.1 (0.2, 9.6) | 0.7 | 0.2 (0,0) | **1** | |
| Random Effects | 1.5 |  |  |  |  |  |  |  | |
| **Complete Cases Model-** Fixed Effects |  |  |  |  |  |  |  |  | |
| No Change in Intention | Reference | **-** | Reference | - | References |  | References |  | |
| Decreased Intention | 0.03 (-0.2, 0.3) | 0.92 | -0.01 (-0.3, 0.2) | 0.9 | 4.3 (0.3, 69.5) | 0.3 | 0 |  | |
| Increased Intention | 0.33 (0.1, 0.6) | **0.01** | 0.2 (-0.03, 0.5) | 0.07 | 0.5 (0.01, 30.4) | 0.7 | 0 |  | |
| Random Effects | 1.5 |  | 1.5 |  | 3.3 |  | 3.3 |  | |
| **Perceived infertility risk** | | | | | | | | |  |
| **Multiple Imputation Model**- Fixed Effects |  |  |  |  |  |  |  |  | |
| No Change in Intention | Reference | - | Reference | - | References |  | References |  | |
| Decreased Intention | 0.02 (-0.3, 0.3) | 0.9 | 0.1 (-0.1, 0.5) | 0.3 | 1.4 (0.5, 3.7) | 0.5 | 2.58 (1.2, 5.7) | **0.02** | |
| Increased Intention | 0.03 (-0.3, 0.4) | 0.8 | 0.2 (-0.2, 0.6) | 0.3 | 1.3 (0.4, 4.0) | 0.6 | 1.12 (0.4, 2.8) | 0.8 | |
| Random Effects | 1.7 |  | 1.5 |  |  |  |  |  | |
| **Complete Cases Model-** Fixed Effects |  |  |  |  |  |  |  |  | |
| No Change in Intention | Reference | **-** | Reference | - | References |  | References |  | |
| Decreased Intention | -0.03 (-0.2, 0.1) | 0.7 | 0.1 (-0.04, 0.3) | 0.13 | 2.4 (0.4, 13.9) | 0.3 | 4.6 (0.9, 25.0) | 0.07 | |
| Increased Intention | 0.06 (-0.1, 0.2) | 0.5 | 0.2 (-0.01, 0.3) | 0.06 | 1.4 (0.2, 10.9) | 0.7 | 0.7 (0.07, 6.3) | 0.7 | |
| Random Effects | 1.7 |  | 1.7 |  | 3.3 |  | 3.3 |  | |
| ^a^ Model adjusted for time, race, ethnicity, age at baseline, education, BMI, general health, stress, social support, perceived infertility risk  ^b^ Model adjusted for time, race, ethnicity, age at baseline, education, employment, household income, BMI, general health, stress, perceived infertility risk  ^c^ Model adjusted for time, age at enrollment, race, ethnicity, education, marital status, employment, income, BMI, general health, presence of insurance, stress, social support, comorbidities, parity, perceived infertility risk  ^d^ Model adjusted for time, race, ethnicity, employment, income, perceived infertility risk | | | | | | | | |  |

**Appendix Table 6.** Mixed effects models of the association of changes in pregnancy intention score (PIS) (left) and trying to become pregnant (right) with physical activity and smoking, stratified by parity

|  | **Physical Activity** | | | | | | | **Smoking** | | | |
| --- | --- | --- | --- | --- | --- | --- | --- | --- | --- | --- | --- |
|  | **PIS^a^** | | | | | **Trying^b^** | | **PIS^c^** | | **Trying^d^** | |
|  | Adjusted  B(95% CI) | | p | | | Adjusted  B (95% CI) | p | Odds Ratio  (95% CI) | p | Odds Ratio  (95% CI) | p |
| **Nulliparous** | | | | | | | | | | | |
| **Multiple Imputation Model**- Fixed Effects | |  | |  |  | |  |  |  |  |  |
| No Change in Intention | | Reference | | - | Reference | | - | References |  | References |  |
| Decreased Intention | | 0.02 (-0.2, 0.3) | | 0.9 | 0.1 (-0.2, 0.4) | | 0.5 | 1.4 (0.5, 3.7) | 0.5 | 2.9 (1.0, 8.6) | 0.05 |
| Increased Intention | | -0.03 (-0.3, 0.3) | | 0.8 | 0.2 (-0.1, 0.5) | | 0.2 | 0.8 (0.2, 2.8) | 0.7 | 0.8 (0.2, 2.8) | 0.7 |
| Random Effects | |  | |  |  | |  |  |  |  |  |
| **Complete Cases Model-** Fixed Effects | |  | |  |  | |  |  |  |  |  |
| No Change in Intention | | Reference | | **-** | Reference | | - | References |  | References |  |
| Decreased Intention | | -0.02 (-0.2, 0.2) | | 0.8 | 0.1 (-0.1, 0.3) | | 0.6 | 1.5 (0.2, 12.7) | 0.7 | 6.9 (0.9, 49.6) | 0.05 |
| Increased Intention | | -0.01 (-0.2, 0.2) | | 0.8 | 0.2 (-0.03, 0.3) | | 0.1 | 0.5 (0.04, 6.3) | 0.6 | 0.2 (0.01, 7.5) | 0.4 |
| Random Effects | | 1.8 | |  | 1.8 | |  | 3.3 |  | 3.3 |  |
| **Parous** | | | | | | | | | | | |
| **Multiple Imputation Model**- Fixed Effects |  | | |  |  | |  |  |  |  |  |
| No Change in Intention | Reference | | | - | Reference | | - | References |  | References |  |
| Decreased Intention | 0.1 (-0.3, 0.5) | | | 0.6 | 0.3 (-0.1, 0.8) | | 0.1 | 1.6 (0.6, 4.5) | 0.3 | 0.8 (0.2, 3.5) | 0.8 |
| Increased Intention | 0.6 (0.1, 1.1) | | | **0.02** | 0.2 (-0.2, 0.6) | | 0.4 | 1.6 (0.4, 6.1) | 0.4 | 0.9 (0.2, 3.7) | 0.8 |
| Random Effects |  | | |  |  | |  |  |  |  |  |
| **Complete Cases Model-** Fixed Effects |  | | |  |  | |  |  |  |  |  |
| No Change in Intention | Reference | | | **-** | Reference | | - | References |  | References |  |
| Decreased Intention | 0.04 (-0.2, 0.3) | | | 0.7 | 0.1 (-0.1, 0.3) | | 0.2 | 4.5 (0.5, 44.7) | 0.5 | 0.7 (0.03, 18.2) | 0.8 |
| Increased Intention | 0.47 (0.2, 0.7) | | | **0.001** | 0.3 (0.03, 0.5) | | **0.02** | 2.6 (0.2, 44.3) | 0.5 | 1.3 (0.1, 20.6) | 0.8 |
| Random Effects | 1.3 | | |  | 1.4 | |  | 3.3 |  | 3.3 |  |
| ^a^ Model adjusted for time, race, ethnicity, age at baseline, education, BMI, general health, stress, social support, perceived infertility risk  ^b^ Model adjusted for time, race, ethnicity, age at baseline, education, employment, household income, BMI, general health, stress, perceived infertility risk  ^c^ Model adjusted for time, age at enrollment, race, ethnicity, education, marital status, employment, income, BMI, general health, presence of insurance, stress, social support, comorbidities, parity, perceived infertility risk  ^d^ Model adjusted for time, race, ethnicity, employment, income, perceived infertility risk | | | | | | | | | | | |
